# Properties of poly (vinyl chloride) membranes containing cadmium pigments, irradiated with UV radiation

**DOI:** 10.1038/s41598-021-96713-w

**Published:** 2021-09-13

**Authors:** Magdalena Tworek, Łukasz Skowroński, Edwin Makarewicz, Joanna Kowalik

**Affiliations:** 1 Faculty of Chemical Engineering and Technology, UTP University of Science and Technology, Seminaryjna 3, 85-326 Bydgoszcz, Poland; 2 Institute of Mathematics and Physics, UTP University of Science and Technology, Kaliskiego 7, 85-796 Bydgoszcz, Poland

**Keywords:** Polymer chemistry, Polymer characterization

## Abstract

The cadmium pigments were yellow, orange and red pigments. They consisted of cadmium sulphide and cadmium sulphide with zinc sulphide as well as cadmium sulphide with cadmium selenide. Their quantitative composition and specific surface area were examined. The pigments were used to color the poly (vinyl chloride) plastisol films, which were then exposed to UV radiation. The surfaces of the coloured membranes were examined by infrared spectroscopy before and after irradiation with UV. The changes occurred in the PVC membrane were investigated by thermogravimetric analysis. The degree of crystallinity of the pigments and the membrane was determined by X-ray diffraction. The color change of the membranes was determined from the spectra obtained by reflection spectroscopy, and the components of colour L*, a* and b* were calculated. Base of them, the tolerance of colour deviations (ΔE*) was determined. The calculations allowed for the determination of the effect of UV irradiation on the change of the colour of the membranes and confirmation of the degradation of the pigments and polymer membrane.

## Introduction

Pigments in paint coatings fulfill decorative and protective functions. Coloring of plastics is one of the factors determining the so-called life cycle of the product made of the material^1^. The most interesting research issue is explaining the effects and causes of the color change of pigments and the properties of polymer products under the influence of UV radiation. The spectral properties of solar radiation for PVC plastisol films with various fillers have been described by Jaoua-Bahloul et al.^2^. Studies have not shown to improve the optical properties of the PVC plastisol containing titanium oxide and pearl. Matuana et al.^3^ examined the weather resistance of titanium dioxide-pigmented composites of polyvinyl chloride with wood fibres. The samples of composites were irradiated with UV lamps. It was found that wood fibres accelerated the degradation of the composite caused by radiation. The durability of the composites increased when titanium dioxide of rutile structure was added to PVC. Bigger, Gribben and Rogers^4^ investigated the change in colour of polyamide products aged in water. The products were coloured with copper complexes and compounds, phthalocyanine blue and cobalt pigments. The change in the colour of polyamide products was caused by thermal degradation products as well as leaching and hydrolysis of copper-containing compounds. Pastorelli et al.^5^ studied the effect of heat, humidity and light on the degradation of polymeric materials in the natural environment. Colour changes of carboxymethyl cellulose, polyamide, polyvinyl chloride, polystyrene, polyethylene, polypropylene, polycarbonate, polyurethane, melamine–formaldehyde and phenol–formaldehyde resins were measured. The results of the colour measurements suggested that the factors which had the greatest impact were light and the mixture of nitrogen oxides. The degradation of materials is influenced to a lesser extent by the concentration of ozone, sulphur dioxide, heat and humidity. Auger and McLoughlin^6^ investigated colour fading of acrylic coatings with titanium white of rutile structure and yellow pigments when exposed to UV radiation. They also studied compositions of white pigments with cadmium yellow and red pigments being mixtures of cadmium sulphide and cerium sulphide. The complex mechanism of CdS yellows photooxidation under the influence of light and moisture was presented in the work of Van der Snickt et al. and Comelli et al.^7,8^. It involves the direct photo-oxidation of cadmium sulphide to cadmium sulphate. Further reactions may lead to the development of other types of cadmium.

Exposure of the PVC polymer to ultraviolet light can release hydrogen chloride (HCl) accompanied by the formation of polyene sequences and cross-links in the chain, causing a rapid degradation process, usually manifested by a color change from yellow to dark brown. This process is known as dehydrochlorination. A simplified mechanism of the PVC dehydrochlorination process was included in the work of Rodolfo and Mei^9^. The results of research on the properties of cadmium pigments after chemical exposure and UV irradiation as well as of polyvinyl chloride plastisol membranes, including their presence before and after UV irradiation, are presented in other studies^10–12^.

In this paper, yellow, orange and red cadmium pigments consisting of cadmium sulphide or their crystalline mixtures with cadmium selenide were selected for research. It is a group of pigments used to color poly (vinyl chloride) plastisols, polymer products and paints whose coatings are resistant to high temperature, chemical agents and solar radiation. The aim of the study was to determine the effects of UV radiation on the properties of colored poly (vinyl chloride) membranes. The scientific goal was to determine the mechanism of polymer and pigment degradation as well as the type of changes and products in the colored films resulting from irradiation. The studies of colored membranes consisted in determining the structural and surface changes formed in the membranes by thermogravimetry and infrared spectroscopy. The chemical composition of pigments and their specific surface were determined. By means of the L*, a* and b* color indicators, the effects of the influence of UV radiation on their change were indicated.

## Experimental part

### Materials

Inorganic pigments received from Chemical Factory *Permedia S.A*. in Lublin (Poland). The examined cadmium pigments were: cadmium yellow ST (CdS), cadmium yellow WO-2 (CdS × 0.2ZnS), cadmium orange SZ-1 (CdS × 0.1CdSe), cadmium orange SW-1 (CdS × 0.2CdSe), cadmium red 88-3 (CdS × 0.35CdSe), cadmium red 88-4 (CdS × 0.5CdSe), cadmium red 88-7 (CdS × 0.55CdSe) and cadmium red 88-71 (CdS × 0.65CdSe). The particle size of the pigments was 2.0–5.0 µm. The above-mentioned names, symbols and quantitative compositions were provided by the manufacturer. The characteristics of the tested pigments were compared to titanium white named Tytanpol R-003 in the form of a white powder with a crystallographic structure of rutile, surface-treated with aluminium and zirconium compounds and hydrophilic organic compounds. The manufacturer of titanium white was the Chemical Company Police S.A., the Azoty Group. For the purification of pigments, *n*-butyl acetate, analytical grade, a product of Avantor Performance Materials Poland S. A. in Gliwice was used. In plastisol, the polymer was emulsion polyvinyl chloride designated with the symbol PVC E-68 P-AAS-d-bs (PVC E-68 Pbs) in the form of a white powder with the number K = 69.5, manufactured by Synthos S. A. in Oświęcim. The plasticiser was di-2-ethylhexyl phthalate (DOP) with the density d = 0.986 g g/cm^3^ and the viscosity of 79.0 mPas at 20 °C. The manufacturer of the plasticiser named Ergoplast FDO was Boryszew Company S. A., Boryszew ERG Branch in Sochaczew.

### Methods

#### Pigments preparation

Pigments were purified by extraction with n-butyl acetate in a Soxhlet apparatus for 2.5 h. The Soxhlet extraction removes surfactant (oleic acid) that was adsorber on the pigment surface. Then, the pigment sample was moved from the chamber of the extraction apparatus onto the Petri dish and dried in a forced air dryer at a temperature of 85.0 °C to constant mass. Each sample of the dried pigment was thoroughly ground in a mortar to a homogeneous powder.

#### Plastisol preparation

Plastisols consisted of 100 parts by mass of PVC and 120 parts by mass of DOP. The compositions were prepared by careful mechanical mixing in a mortar of PVC powder with a plasticizer for 8 h. The plastisols were vented at rest in a HZV vacuum dryer at a pressure of 6.5 hPa and at 22.0 °C for 96.0 h. Samples were used for the tests after 24 h from the end of venting. The plastisols were homogeneous, did not stratify and did not contain sediment. The exact method of making plastisols is given in monographs^13,14^.

#### Colored membranes preparation

The pigments were introduced into plastisols at an amount of 14.3 wt%. The preparation of coloured plastisols consisted of weighing 24.0 g of plastisol samples in porcelain mortars and adding 4.0 g of powdered pigment to each of them. The content of each mortar was ground for 2.0 h until the material was homogeneous and uniform in colour. The coloured plastisols were deaerated again in a vacuum dryer for 24 h. The deaerated plastisols were thoroughly mixed and poured into glass Petri dishes with a diameter of 15.0 cm. The dishes were placed in a dryer at 140 °C with forced air circulation for a period of 1 h to obtain gel. After the set gel time had elapsed, the dishes were removed from the dryer and cooled down under room conditions. Coloured membranes with a thickness of approx. 1.0 mm were obtained and tested.

### Characterization techniques

#### UV exposure of samples

The irradiation of the coloured membrane samples was performed in a Q-SUN Xe-1B apparatus equipped with a xenon lamp. The membrane samples were in the form of 40 × 100 mm stripes. They were marked and attached to a cardboard base. The temperature in the chamber was 60 °C. Irradiation was carried out for 20 h per day. The total exposure time of the samples in the apparatus was 100 h^15^.

#### X-ray energy dispersion

The X-ray fluorescence energy dispersion method was used to determine the qualitative and quantitative composition of pigments. The tests were performed using the MiniPal PW4025/00 X-ray energy dispersion spectrometer. The method was based on the fact that each element contained in the analyzed sample, due to X-ray excitation, emitted a spectrum that was characteristic of it, which was the basis for qualitative and quantitative analysis^16–19^.

#### Adsorption

The adsorption studies were aimed at determining the size of the specific surface area of pigments. Methylene blue (BM) and acid orange II (OK II) were used as the adsorbate. The tests were performed using the static method of dye water solution at room temperature. They consisted in mixing 0.2 g of the tested pigment with 0.1 dm3 of BM or OKII solution with variable concentration ranging from 1.0 to 42.0 mg/dm^3^. The experiment time, ensuring the establishment of the adsorption equilibrium, was 30 min and was determined from the previous kinetic measurements. The concentration of BM or OK II adsorbate in the solution before and after adsorption was measured spectrophotometrically at a wavelength of 665 nm for BM and 484 nm for OK II. The tests were carried out using a Merck Pharo 300 spectrophotometer. The molecular weight of the methylene blue was 320 g/mol and the acid orange II was 350.32 g/mol. The area occupied by one molecule of methylene blue was 135, 10–20 m^2^ and for acid orange II 120, 10–20 m^2^. The detailed method of carrying out adsorption studies and the description of isotherms using the linearized form of the Langmuir equation, as well as the calculation of the specific surface area of pigments, was performed in accordance with the methodology described in the works^20–23^.

#### Thermal gravimetric analysis (TG-DTG) and differential thermal analysis (DTA)

Thermogravimetric tests of samples of coloured PVC membranes were performed using a Q1500D derivatograph of the Paulik–Paulik–Erdey system provided by Hungarian MOM company in the air atmosphere. Ceramic crucibles and 200 mg samples were used. The weight loss of the sample was recorded continuously as the temperature increased to 1000 °C at the rate of 5.0 °C/min. Thermal effects of the thermal decomposition of membranes were calculated based on the DTA curve in relation to a standard,which was benzoic acid^24,25^.

#### Infrared spectrometry

FTIR spectroscopic examinations of coloured PVC membranes were performed with the use of a Bruker α-Alpha-P device with a reflective diamond head and Opus 6.5 software. The measurement was carried out in the wave number range of 350–4000 cm^−1^. The measurement was recorded with a resolution of 4.0 cm^-1^. Characteristic maxima for selected groups appearing in the spectra were provided based on the monograph and presented in Table 1^26–29^. The atoms or groups present in the spectrum were characterised using their wave number and transmittance value.

**Table 1:** Wave number ranges used to interpret test results.

Wavenumber range, cm^−1^	Vibration type	The type of bond or moiety and its intensity
1: 3200–3700	Valence	O–H alcohols with hydrogen bridges, monomers of carboxylic acids (medium and broad bands), carboxylic acids with hydrogen bridges (broad bands)
2: 450–800	Stretching	C–Cl, CH mainly from the CH–Cl moiety

#### X-ray diffraction analysis (XRD)

Roentgenographic examinations were performed with a Seifert URD 6 X-ray diffractometer with a goniometer using CuKα radiation and a nickel filter. The degree of crystallinity of pigments and coloured membranes was calculated as the ratio of the area of the crystalline to the amorphous surface area in the range of 2 theta 10–50^30,31^.

#### Colour change determination

The colour of the membrane samples was measured using a Varian Cary 5000 spectrophotometer. The reflectance spectra in the range from 200 to 800 nm were recorded. In order to describe the course of the obtained curves in the CIELAB colour space, the coordinates of the L*, a* and b* indices were calculated according to the method presented in other studies^32,33^. To determine the difference between the colours of the samples before and after its exposure to UV radiation, the parameter ΔE* called the tolerance of colour deviation criterion was used. The tolerance of colour deviation can be calculated according to the dependencies provided in other studies^32–35^:

1 $$\Delta {{E_{1,2}}^{*}} = \sqrt {\left( {{{L_{1}}^{*}} - {{L_{2}}^{*}} } \right)^{2} + \left( {{{a_{1}}^{*}} - {{a_{2}}^{*}} } \right)^{2} + \left( {{{b_{1}}^{*}} - {{b_{2}}^{*}} } \right)^{2} } ,$$

where L_1_*, a_1_* and b_1_* were the values of the membrane colour components before UV irradiation, while L_2_*, a_2_* and b_2_* were the values of the membrane colour components after UV irradiation.

## Results and discussion

### Composition of pigments

The cadmium pigments used for the study were industrial products. Their exact elemental compositions were determined by X-ray fluorescence analysis. Table 2 presents the results of tests of the compositions of individual pigments calculated on the basis of their formulas provided by the manufacturer and determined by the analytical method.

**Table 2:** Contents of elements in cadmium pigments.

Pigment	M_c_	Composition calculated, wt%			Composition marked, wt%		
		Cd/Zn	S	Se	Cd/Zn	S	Se
Cadmium yellow ST	144	77.78	22.22	–	79.25	20.75	–
Cadmium yellow WO-2	163.4	68.54/7.96	23.50	–	33.90/55.70	10.40	–
Cadmium orange SZ-1	163.1	75.54	19.62	4.84	76.80	18.50	4.70
Cadmium orange SW-1	182.2	73.77	17.56	8.67	73.60	17.00	9.40
Cadmium red 88-3	210.85	71.71	15.18	13.11	76.60	11.30	12.10
Cadmium red 88-4	239.5	70.15	13.36	16.49	75.40	13.10	11.50
Cadmium red 88-7	249.05	69.70	12.85	17.45	75.20	8.90	15.90
Cadmium red 88-71	268.15	68.92	11.93	19.15	74.00	8.60	17.40

The data presented in Table 2 demonstrate that there were differences in the amounts of individual elements between the amount calculated on the basis of the pigment chemical composition provided by the manufacturer and the amount determined by the X-ray fluorescence method. The biggest difference was recorded for cadmium yellow WO-2, in which, according to of the manufacturer, the amount was approx. 8.0 wt% of zinc, and the determined amount of this element was approx. 56.0 wt%. In the case of the remaining elements, the differences between the calculated amount of elements and the determined composition was approx. 5.0%. The study confirmed the presence of differences in the amounts of individual elements in pigments. Most probably, this was caused by the different technological conditions of their production. In Table 3 the determined values of the specific surface area of the pigments are presented.

**Table 3:** Values of specific surface of cadmium pigments, determined by means of methylene blue and acidic orange II.

Type of pigment	Methylene blue S × 10^3^m^2^/g	Acid orange II S × 10^3^m^2^/g
Cadmium yellow ST	5.22	5.60
Cadmium yellow WO-2	3.78	3.55
Cadmium orange SZ-1	5.44	4.15
Cadmium orange SW-1	2.72	1.95
Cadmium red 88-3	9.00	7.26
Cadmium red 88-4	5.21	4.72
Cadmium red 88-7	2.70	3.45
Cadmium red 88–71	6.71	5.18

All the test pigments were characterised by a small specific surface area. Their values varied greatly. It can be assumed that the value of the pigment specific surface area was undoubtedly influenced by the method of its production. The ions of the positively charged dye cation were attracted by electrostatic force to negatively charged sites on the surface of the pigments. This proved that there are anionic adsorption centres on the surface of these pigments. In order to determine positively charged sites on the surface of the pigments, tests with the use of acid orange II, which formed dye anions in water, were conducted. It was observed that the size of the specific surface area of the pigments determined with the use of acid orange II was comparable to the area determined with the use of methylene blue. This meant that on the surface of the pigment particles there was a similar number of sites that were centres of negative and positive charge adsorption activity.

### Infrared spectrometry

**Figure 1:**
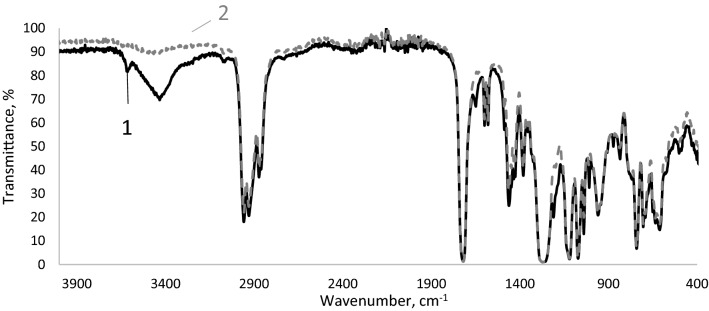
Full-infrared spectrum of PVC plastisol membranes containing cadmium yellow (CdS) before (1) and after (2) UV irradiation.

The test pigments were used to colour polyvinyl chloride plastisols. The problem was to explain their influence on the characteristics of the membranes obtained by gelation of PVC plastisol. The changes caused by the UV irradiation of the membrane surfaces were examined spectroscopically over the full range of the IR spectrum. In Fig. 1 IR spectra of PVC membranes containing cadmium yellow ST are presented.

**Figure 2:**
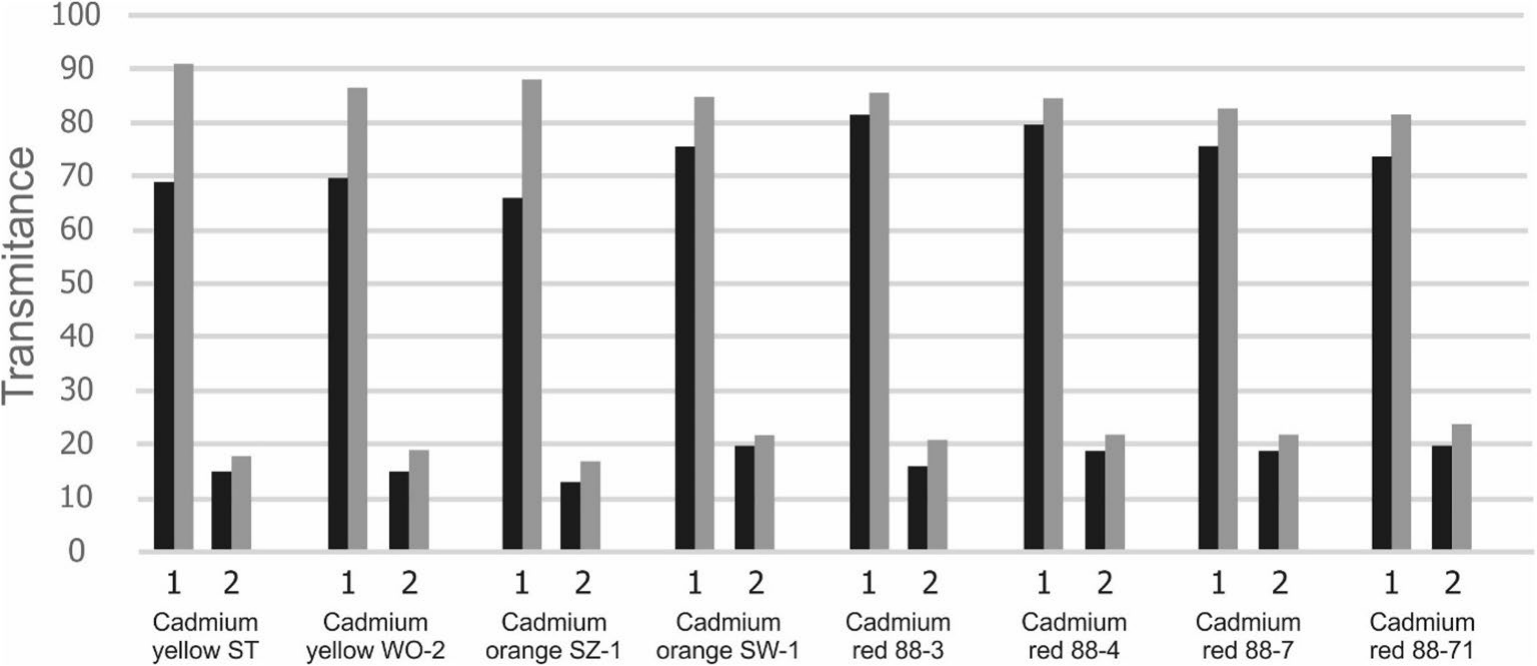
Transmittance values of PVC films containing pigments before and after UV irradiation Explanations: 1,2—wave number ranges given in Table 1.

The analysis of the spectra presented in Fig. 1 showed that the most significant changes in transmittance occurred in the wave number ranges specified in Table 1. Figure 2 shows the results.

It can be concluded that in the first wave number measuring range (Table 1) the transmittance concerned the presence of hydroxyl groups. Most probably they were part of the plasticiser. Exposing the membranes to UV radiation caused an increase in transmittance, or a reduction in the number of hydroxyl groups. The second spectral range was characterised by the presence of chlorine atoms in the membrane. Lower transmittance values indicated their higher content in the polymer. However, as a result of irradiation, dehydrochlorination took place. This phenomenon was confirmed by higher transmittance values. The membrane was made of polyvinyl chloride plasticised with di-2-ethylhexyl phthalate. The resulting hydrogen chloride reacted with the pigment. The increase in transmittance depended on the type of the applied pigment. In the case of yellow and orange pigments, there are greater changes in transmittance. Yellow pigments contain readily reacting cadmium sulphide. Smaller transmittance changes were found in pigments with an increased amount of cadmium selenide. Overall, a very small effect of the type of pigment on the dehydrochlorination of the polymer can be found.

### Thermal stability of coloured film

Yellow pigments contain easily reacting zinc sulphide. Smaller transmittance changes were found in pigments with an increased amount of cadmium selenide. A very small effect of the type of pigment on polymer dehydrochlorination was observed.

**Figure 3:**
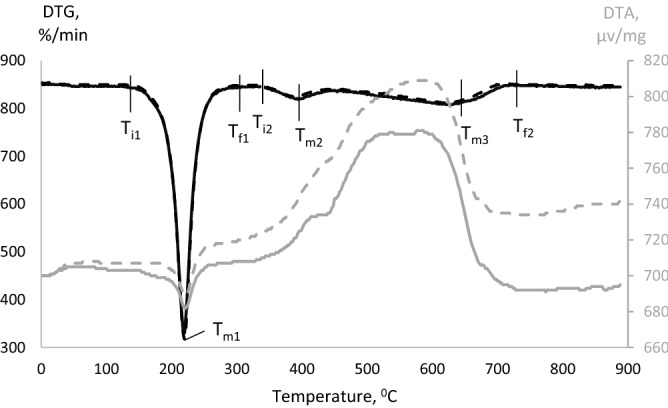
Thermal gravimetric graphs and differential thermal analysis graphs for a sample of PVC film containing purified ST cadmium yellow before (black line) and after (black dashed line) UV irradiation.

The coloured membranes were subjected to thermogravimetric analysis. The results were obtained in the form of DTG, DTA and TG curves. On their basis, the types of thermal transformations presented as stages of decomposition were analysed. For each pigment, the initial and maximum decomposition rates and final transformation temperatures were determined. Corresponding weight percent changes and thermal effects were calculated. In Fig. 3, exemplary DTG and DTA curves for a membrane with cadmium yellow ST are presented.

The temperatures describing the changes taking place in the membrane during its thermal decomposition were determined based on the thermogravimetric curves. The course of the curve indicated a decomposition of the membrane in two steps. In Table 4, the results of thermogravimetric analysis of the membranes prior to their exposure to UV radiation were presented. The TG-DTG-DTA diagrams of the other membranes are included in the Supplementary Appendix.

**Table 4:** PVC membranes with cadmium pigments.

Pigment	Thermal decomposition stage	Initial temperature,Ti_1,_T_i2_, °C	Temperature of the maximum rate of mass change T_m1_, T_m2_, T_m3_, °C		Final temperature, T_f1_, T_f2_, °C	Total weight loss in the 1st and 2nd stage, wt%	Thermal effect, kJ/g
PVC film	1	194.0	294.0		381.0	81.0	− 0.15
	2	401.0	593.0		703.0	100.0	3.22
Cadmium yellow ST	1	189.0	278.0		344.0	64.0	− 0.17
	2	376.0	661.0		792.0	94.0	4.34
Cadmium yellow WO-2	1	185.0	235.0		327.0	59.0	− 0.14
	2	391.0	587.0	740.0	812.0	91.0	4.71
Cadmium orange SZ-1	1	197.0	275.0		348.0	67.0	− 0.22
	2	397.0	482.0	662.0	815.0	92.0	3.53
Cadmium orange SW-1	1	187.0	282.0		368.0	66.0	− 0.24
	2	386.0	477.0	627.0	771.0	87.0	2.61
Cadmium red 88-3	1	202.0	289.0		384.0	72.0	− 0.14
	2	395.0	488.0	633.0	735.0	93.0	3.53
Cadmium red 88-4	1	207.0	284.0		381.0	68.0	− 0.13
	2	411.0	486.0	626.0	784.0	86.0	3.14
Cadmium red 88-7	1	205.0	285.0		385.0	68.0	− 0.12
	2	403.0	493.0	615.0	737.0	86.0	2.93
Cadmium red 88-71	1	181.0	291.0		391.0	69.0	− 0.14
	2	403.0	481.0	588.0	673.0	86.0	2.47

Based on the data presented in Table 4, in the first stage, the initial temperature of membrane decomposition was higher than in the case of membranes containing pigments. The membrane with cadmium red 88-71, with the highest content of cadmium selenide, had the lowest value of the initial temperature. Moreover, the value of the maximum temperature of the weight change rate of the sample was the highest when the membrane did not contain pigments. In turn, the lowest value of this temperature corresponded to the membrane containing cadmium yellow with zinc sulphide WO-2. The final degradation temperatures of the membranes varied considerably. The membranes with cadmium yellow with zinc sulphide had the lowest temperature. The highest final temperature corresponded to the cadmium red 88-71 membrane with the highest content of cadmium selenide. It occurred that after the end of the test, the presence of the pigment in the membrane contributed to the formation of a larger amount of ash residue. At this stage of degradation, the thermal effects have always been endothermic. In the second stage of decomposition, the membrane with cadmium red 88-71 had the highest value of the initial decomposition temperature. The membrane without pigment and with cadmium yellow had the highest temperature value of the maximum rate of weight change. At this stage of membrane decomposition, a second temperature of the maximum rate of change in sample weight was found. Most probably, its value corresponded to the decomposition of the formed intermediate products that could be a result of the decomposition of the polymer or the plasticiser with the pigment. In such cases, the final decomposition temperature was higher and more ash was produced. In the second stage of decomposition, the thermal effect was exothermic and was the smaller the more cadmium selenide in the pigment. The study of thermal decomposition of membranes showed that due to different chemical compositions of pigments, each case should be considered individually. In Table 5, the results of thermal decomposition of PVC membranes containing purified cadmium pigments after irradiation were shown.

**Table 5:** PVC membranes with cadmium pigments after UV irradiation.

Pigment	Thermal decomposition stage	Initial temperature,T_i1_, T_i2_, °C	Temperature of the maximum rate of mass change T_m1_, T_m2_,T_m3_, °C		Final temperature,T_f1_ , T_f2_, °C	Total weight loss in the 1st and 2nd stage, wt%	Thermal effect, kJ/g
PVC film	1	187.0	291.0		372.0	79.0	− 0.09
	2	401.0	563.0		681.0	99.6	3.14
Cadmium yellow ST	1	188.0	275.0		343.0	61.0	− 0.14
	2	398.0	649.0		795.0	93.0	4.24
Cadmium yellow WO-2	1	184.0	238.0		321.0	58.0	− 0.08
	2	418.0	582.0	759.0	813.0	93.0	4.94
Cadmium orange SZ-1	1	196.0	275.0		349.0	64.0	− 0.16
	2	405.0	478.0	656.0	786.0	90.0	3.65
Cadmium orange SW-1	1	197.0	283.0		356.0	65.0	− 0.16
	2	409.0	472.0	655.0	823.0	89.0	3.74
Cadmium red 88–3	1	200.0	288.0		376.0	70.0	− 0.12
	2	413.0	483.0	620.0	786.0	92.0	3.63
Cadmium red 88-4	1	213.0	285.0		380.0	66.0	− 0.15
	2	402.0	483.0	627.0	757.0	85.0	3.41
Cadmium red 88-7	1	207.0	291.0		387.0	66.0	− 0.16
	2	410.0	472.0	621.0	753.0	85.0	4.23
Cadmium red 88-71	1	216.0	286.0		367.0	65.0	− 0.11
	2	412.0	463.0	590.0	718.0	81.0	3.34

Based on the data presented in Table 5, it can be concluded that the values of the initial and maximum rate of weight change and final temperature after the first and second stages of membrane decomposition changed. They consisted in its slight increase or decrease. The colourless membrane behaved differently after irradiation. The initial temperatures of the first and second decomposition stages slightly decreased. The temperatures of the maximum rate of change in sample weight were virtually the same. Final temperatures underwent a slight decrease. In the first stage, the initial decomposition temperatures of the membrane with cadmium yellow ST were the same. They were higher only in the second stage. In the first stage of membrane decomposition, the temperature of the maximum rate of weight change was slightly decreased and in the second stage it was almost the same. In the case of the final temperatures, it can be concluded that if, when exposed to radiation, only degradation of the membrane related to the reduction of its molecular weight took place, then the final temperature had lower values. However, if the macroradicals formed during the degradation of the polymer underwent recombination reactions leading to cross-linking of the polymer, then a higher final temperature was observed. In the course of the discussed reactions, pigments could contribute to the formation of new products. This was confirmed by the second stage of membrane decomposition in which the second temperature value of the maximum rate of weight change and greater thermal effects occurred. The total weight losses of the sample was very similar.

### Crystal structure

X-ray examinations were performed to determine the degree of crystallinity of pigments and coloured membranes before and after their exposure to UV radiation. In Table 6 their values were presented.

**Table 6:** Values of crystalinity degree of cadmium pigments and membranes with cadmium pigments before and after exposure to UV radiation.

Pigment	The degree of pigment crystallinity, %	The degree of crystallinity of the film with pigment before UV exposure, %	The degree of crystallinity of the pigmented film after UV irradiation,%
Cadmium yellow ST	92.8	34.8	32.9
Cadmium yellow WO-2	117.8	24.5	46.6
Cadmium orange SZ-1	120.3	39.3	29.3
Cadmium orange SW-1	128.1	37.8	38.6
Cadmium red 88-3	100.7	30.9	31.2
Cadmium red 88-4	100.1	40.8	36.1
Cadmium red 88-7	90.0	35.4	40.6
Cadmium red 88-71	91.7	29.3	35.5

The values of the degree of crystallinity of the pigments and coloured membranes before and after irradiation specified in Table 6 differed significantly. Cadmium reds 88-7 and 88-71 with the highest content of cadmium selenide had the lowest values of the degree of crystallinity. Slightly higher values were characteristic for cadmium yellow ST and cadmium reds 88-4 and 88-3, while the remaining yellow and orange pigments had similar values of the degree of crystallinity. The analysis of the degree of crystallinity values of the membranes before and after UV irradiation showed that the type of pigment and the exposure significantly influenced the values of the degree of crystallinity. A comparison of the diffraction pattern of the membrane with the diffraction patterns of its components, revealed that the degree of crystallinity of the polymer in the membrane and its structure generally remained in the same spectral position. The degree of crystallinity of the PVC plastisol membrane was 48.9%. Based on the diffractometric analysis of the coloured membranes, it could be concluded that the peaks corresponding to the pigment were shifted towards the reduction of the interplanar spaces. In most cases, the degree of crystallinity of the coloured and irradiated membranes was clearly lower than in the case of the pure polymer membrane. It was found that there was no correlation between the degree of crystallinity of the pigment and the membrane. The gelation process of PVC plastisol at elevated temperature and exposure to UV radiation generally resulted in a reduction in the degree of membrane crystallinity as a result of various decomposition and degradation reactions, and sometimes reactions between the formed products as well as cross-linking. The increase in the degree of crystallinity of the colored films after their irradiation proves the course of the processes of creating new crystalline substances as well as the formation of a cross-linkage network between polymer macro chains. They can also be reaction products derived from the degradation of the polymer with the pigment.

### Reflection spectrophotometry

**Figure 4:**
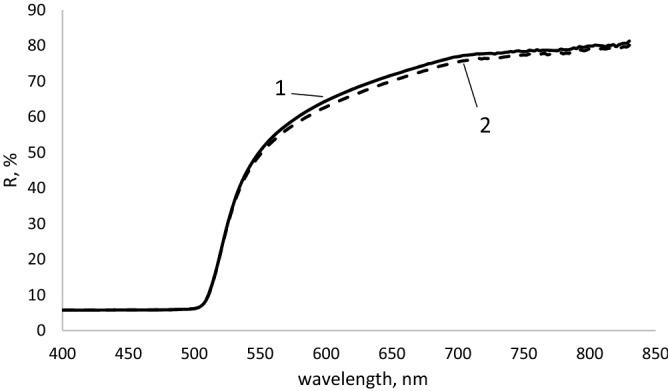
The reflection spectrum of PVC films containing cadmium yellow (CdS) before (1) and after (2) UV irradiation.

An important indicator confirming the influence of UV radiation was the change in the colour of the membranes. The membrane colour measurements were performed before and after UV irradiation. For this purpose, reflectance spectrophotometry was used. It allows for the calculation of colour constants L*, a* and b*. In Fig. 4, the reflectance spectra of PVC membranes containing cadmium yellow ST before and after UV irradiation are presented.

L*, a*, b* coordinates were determined in order to describe the color in CIELab more accurately. Table 7 shows the calculated L*, a* and b values of membranes containing purified cadmium pigments and titanium white before and after UV irradiation.

**Table 7:**
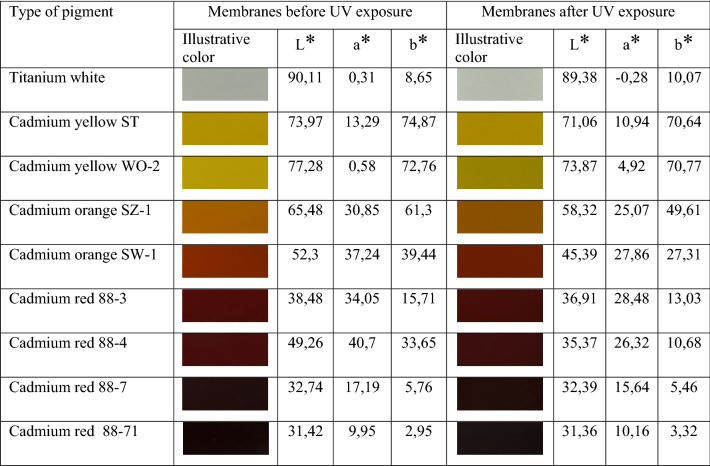
Values of color constants L*, a* and b* for poly (vinyl chloride) plastisol membranes with titanium white and cadmium pigments before and after UV irradiation.

Cadmium pigments consist of cadmium sulphide and selenide. In Table 7 cadmium pigments according to the increasing amount of cadmium selenide in the pigment are presented. The exceptions include titanium white, which is titanium dioxide, and cadmium yellow WO-2, consisting of cadmium sulphide and zinc sulphide. Based on the obtained spectra, all three components describing the colour of the membrane were calculated. The values of the L* component define the luminance of the membranes. The highest values of luminance are characteristic for the membranes containing titanium white and cadmium yellow WO-2. Membranes containing cadmium yellow ST, cadmium orange and cadmium red 88-4 can also be included in this category. The other membranes with red pigments are classified as dark membranes. In particular, these were membranes with cadmium red 88-71. In these membranes, the red pigment has a purple hue. The value of the a* component indicates a shift of the membrane colour towards red or green. The data presented in Table 7 indicate that red and orange pigments have the highest values of the a* component. A similar value of the a* component is demonstrated by cadmium yellow ST and cadmium red 88-71. However, in the case of titanium white and cadmium yellow WO-2, there was a shift towards green. The value of the b* components indicates whether the colour of the membrane is closer to yellow or blue. The data presented in Table 8 show that the values of the b* component are the highest for the yellow and orange pigments as well as cadmium red 88–4. In the case of the remaining pigments, their similarity to blue appeared. After the exposure of the membranes with cadmium pigments to UV radiation, a decrease in the value of all three components of their colour was observed. The values of the L* component decreased the most in the case of the yellow and orange pigments as well as cadmium reds 88-3 and 88-4. In the remaining cases, the luminance of the membranes virtually did not change. The value of the a* component also changed. In the case of the membranes with red pigments, this value decreased. This meant that their red colour was weakened and a shift towards green took place. In the case of the membranes with orange pigments, their red colour was intensified. The membranes with cadmium yellow ST and titanium white virtually did not change. The values of the b* component also changed after the exposure of the membranes to UV radiation. In the case of the membranes with red pigments, there was a decrease in this value, or a colour shift towards blue took place. However, in the case of the membranes with yellow and orange pigments, an increase in the b* component, which meant intensification of colour towards yellow was observed. In contrast, the membranes with titanium white turned yellow. Based on the formula (1), the tolerance values for colour deviations were calculated for all the coloured membranes and pigments. In Table 8, the calculated values of the tolerance values for colour deviations are presented.

**Table 8:** Colour deviation tolerance values for coloured PVC membranes, taking into account the influence of UV radiation.

Pigment	Color deviation tolerance value, ΔE_1,2_*
Titanium white	3.09
Cadmium yellow ST	1.49
Cadmium yellow WO-2	18.47
Cadmium orange SZ-1	1.31
Cadmium orange SW-1	0.92
Cadmium red 88-3	2.34
Cadmium red 88-4	33.39
Cadmium red 88-7	0.89
Cadmium red 88-71	0.68

The analysis of these values leads to the conclusion that the minimum deviations of colour tolerances occur in the case of red pigments 88-7 and 88-71 as well as orange pigments SW-1 and SZ-1 and yellow pigments ST. In the case of red 88-3 and titanium white, a greater influence of UV radiation on membranes can be observed. UV radiation has the greatest influence on the membranes with yellow WO-2 and red 88-4.

## Conclusions

Based on the results of the analysis of the composition of cadmium pigments, significant differences in the content of individual elements were found. The adsorption study showed that all pigments were characterised by a small specific surface area and a comparable amount of positive and negative active adsorption sites. Infrared spectroscopy revealed that the coloured polyvinyl chloride membranes underwent degradation after UV irradiation. The process consisted of dehydrochlorination, polymer breakdown and the reaction of the breakdown products with the pigment. The decomposition products were cadmium chloride and cadmium selenide. Thermogravimetric tests showed the thermal decomposition of the coloured films in two stages. The assumed hypothesis indicating the influence of atoms constituting the pigments on the destruction reaction of the polymer film has been confirmed. The membranes containing pigments with a higher content of cadmium selenide underwent thermal degradation to a lesser extent. The exposure of the membrane to UV radiation changed the temperature of phase transitions, which confirmed the decomposition of the polymer as well as the course of oxidation, cross-linking and recombination reactions. Evidence of the recombination process is the breakdown of membranes in two steps. There was no correlation between the degree of crystallinity of the pigment and the film. The process of gelation of PVC plastisol and irradiation with UV radiation resulted in a reduction in the degree of crystallinity of the films. It was related to the destruction of the polymer and the degradation of the pigments. Exposing the membranes to UV radiation reduced the value of all three color components. This indicated the destructive effect of UV radiation on pigments and the polymer film. Yellow and orange pigments containing less cadmium selenide were more easily decomposed by color change under the influence of UV radiation. The color tolerance deviation values confirmed the greater resistance of membranes with pigments containing the most cadmium selenide.

## Supplementary Information

Supplementary Information.
