# Supplementary material for: Properties of poly (vinyl chloride) membranes containing cadmium pigments, irradiated with UV radiation
Source: Sci Rep. 2021 Sep 13;11:18165. doi: 10.1038/s41598-021-96713-w (PMC8438015; doi:10.1038/s41598-021-96713-w)
Supplement: Supplementary file 1 — Supplementary Information. [file 41598_2021_96713_MOESM1_ESM.docx]

Appendix:

*Thermal gravimetric graphs and differential thermal analysis graphs for a sample of PVC film containing pigments before ( ) and after(---)UV irradiation*

|  |  |
| --- | --- |
|  |  |
|  |  |
|  |  |
